# Inhibition of methane oxidation by nitrogenous fertilizers in a paddy soil

**DOI:** 10.3389/fmicb.2012.00246

**Published:** 2012-07-04

**Authors:** M. Saiful Alam, Zhongjun Jia

**Affiliations:** ^1^State Key Laboratory of Soil and Sustainable Agriculture, Institute of Soil Science, Chinese, Academy of Sciences, Nanjing, Jiangsu ProvinceP.R. China; ^2^Graduate School of Chinese Academy of SciencesBeijing, P.R. China

**Keywords:** methane oxidation, nitrogenous fertilizers, particulate methane monooxygenase gene pmoa, nitrification activity, paddy soil

## Abstract

Nitrogenous fertilizers are generally thought to have an important role in regulating methane oxidation. In this study, the effect of ammonium on methane oxidation activity was investigated in a paddy soil using urea at concentrations of 0, 50, 100, 200, and 400 μg N per gram dry weight soil (N/g.*d.w.s*) and ammonium sulfate at concentrations of 0, 50, and 200 μg N/g.*d.w.s*. The results of this study demonstrate that urea concentrations of 200 μg N/g.*d.w.s*. and above significantly inhibit methane oxidation activity, whereas no statistically significant difference was observed in methane oxidation activity among soil microcosms with urea concentrations of less than 200 μg N/g.*d.w.s* after incubation for 27 days. Similar results were obtained in a sense that methane oxidation activity was inhibited only when the ammonium sulfate concentration was 200 μg N/g.*d.w.s* in soil microcosms in this study. Phylogenetic analysis of *pmoA* genes showed that nitrogen fertilization resulted in apparent changes in the community composition of methane-oxidizing bacteria (MOB). Type I MOB displayed an increased abundance in soil microcosms amended with nitrogenous fertilizers, whereas type II MOB dominated the native soil. Furthermore, although no statistically significant relationship was observed between *pmoA* gene and *amoA* gene abundances, methane oxidation activity was significantly negatively correlated with nitrification activity in the presence of urea or ammonium sulfate. Our results indicate that the methane oxidation activity in paddy soils might be inhibited when the concentration of ammonium fertilizers is high and that the interactions between ammonia and methane oxidizers need to be further investigated.

## Introduction

Methane (CH_4_), a potent greenhouse gas (GHG), is involved in a number of chemical and physical processes in the earth's atmosphere, including global warming (Crutzen, 1995). Despite a short residence time in the atmosphere (10 years), the ability of CH_4_ to absorb infrared radiation is 20–30 times greater than that of CO_2_ (Rodhe, 1990). CH_4_ is more abundant in the Earth's atmosphere now than at any time in at least the past 650,000 years (IPCC, 2007). It has been estimated that 70% of the CH_4_ annually released into the atmosphere is due to human activities including agriculture, waste disposal, and biomass burning (Houghton et al., 2001). During most of the last 150 years, atmospheric CH_4_ has increased monotonically. However, even though the CH_4_ concentration in the atmosphere became erratic and did not increase overall from 1999 until 2007, it has begun to increase again (Rigby et al., 2008).

Paddy fields are an important source of atmospheric CH_4_, contributing approximately 40 Tg year^−1^ (Lelieveld et al., 1998; Wang et al., 2004). CH_4_, which is produced in the soil, enters the roots of the rice plants and is transported through the gas vascular system of the plants to the atmosphere, and oxygen is transported from the atmosphere into the roots. Thus, rice roots are partially oxic, which allows methane-oxidizing bacteria (MOB) to be active in the rhizosphere (Conrad, 2004). MOB associated with the rhizosphere of rice plants oxidize CH_4_ aerobically and use it as a source of carbon and energy. Thus, MOB play an important role in the global CH_4_ budget by reducing CH_4_ emissions from rice ecosystems to the atmosphere (Groot et al., 2003).

When considering the world's continued population growth and economic prosperity, rice production must increase by 40% by 2030 to satisfy the growing demand without adversely affecting the resource base (Khush, 2005). The increasing demand for rice has led to the intensified application of nitrogenous fertilizers, which may affect CH_4_ oxidation because MOB are generally thought to be inhibited by ammonium-based fertilizers, as has been demonstrated for soils (Steudler et al., 1989; King and Schnell, 1994; Gulledge et al., 1997) and sediments (Bosse et al., 1993; Van der Nat et al., 1997). This outcome is at least partially due to the strong evolutionary links between the genetics of enzymes responsible for CH_4_ and ammonia oxidation, which allow MOB and ammonia-oxidizing bacteria (AOB) and/or ammonia-oxidizing archaea (AOA) to switch substrates (Dunfield and Knowles, 1995). This mechanism is believed to be responsible for the inhibition of CH_4_ uptake in soils exposed to high concentrations of available N (Hanson and Hanson, 1996).

In contrast, numerous studies have demonstrated that the activity and growth of MOB in the root zone of rice plants are stimulated upon fertilization (Bodelier et al., 2000a,b; Krüger et al., 2002; Krüger and Frenzel, 2003; Mohanty et al., 2006). This stimulating effect may be the result of a relief in N-source limitation or a direct stimulation of CH_4_ oxidation by NH^+^_4_-N by an as yet unknown mechanism (Bodelier et al., 2000a), and a schematic overview of the general mechanisms by which N-fertilization can influence CH_4_ production and oxidation in wetlands and uplands has been proposed by Bodelier (2011). It is noteworthy to mention that some studies have also demonstrated no effects of ammonium-based N-fertilization on MOB (Dunfield et al., 1995; Delgado and Mosier, 1996; Dan et al., 2001). Even though the effects of nitrogenous fertilizers on CH_4_ oxidation have been the most investigated regulating factor of aerobic CH_4_ oxidation (reviewed in Bodelier and Laanbroek, 2004; Aronson and Helliker, 2010), it is obvious that the interactions between the nitrogen and methane cycle are complex and far from completely understood (Bodelier, 2011). Therefore, microcosm incubations were performed to investigate the effects of urea and ammonium sulfate on methane oxidation in a paddy field to better understand the interactions between the methanotrophic community and ammonia-oxidizing prokaryotes.

## Materials and methods

### Soil

The soil used in this study was collected from a long-term field fertilization experiment at the Changshu Ecological Experimental Station of the Institute of Soil Science, Chinese Academy of Sciences. The paddy soil was developed from lake sediment and is classified as a Typic Haplanthrept based on U.S. soil taxonomy. A full description of this long-term fertilization experiment and its management regimes has been previously described (Wu et al., 2011). A rice–wheat rotation system was maintained in the field, and soil sampling was performed from triplicate plots that received chemical fertilizers (NPK) during the rice-growing season. Bulk soil (top 0–5 cm) was collected and transported on ice to the laboratory immediately after sampling.

### Microcosm incubation

Five grams of fresh soil was placed into a 120 ml crimp top serum vial, and treatments of 0, 50, 100, 200, and 400 μg urea-N/*g d.w.s* or 0, 50, and 200 μg (NH_4_)_2_SO_4_-N/*g d.w.s* were established in duplicate. For the soil microcosms amended with (NH_4_)_2_SO_4_, 388 μg Na_2_CO_3_-C /*g d.w.s* was also added as the carbon source for ammonia oxidizers. All soil microcosms contained slurries with a final volume of 50 ml through the addition of sterile distilled water. The bottles were then sealed with rubber stoppers, and CH_4_ was injected into the headspaces to generate the targeted methane concentration of ~5,000 parts per million. The incubation of soil microcosms was performed at 28°C in the dark with shaking at 200 rpm for 27 days. After consumption of >95% of the CH_4_, the vials were flushed with air to remove any CO_2_ and to ensure that the slurries remained aerobic. The treatments were then renewed after 0, 4, 8, 12, 16, 20, and 24 days of incubation, providing the targeted concentrations of CH_4_ and nitrogenous fertilizers described above. CH_4_ concentrations were measured on a daily basis or every other day, and inorganic nitrogen (NO^−^_3_, NO^−^_2_, and NH^+^_4_) concentrations were determined at days 0, 15, and 27.

Gas samples were collected to determine the CH_4_ concentration in the headspace of the microcosms. Before gas samplings, the bottles were gently shaken by hand for 1 min to release the CH_4_ dissolved in the submerged water layer into the headspace. One milliliter of the gas sample in the headspace was analyzed using gas chromatography with a flame ionization detector, as described previously (Liu et al., 2011). Soil slurries were collected after 0, 15, and 27 days of incubation. Before sampling, the bottles were vigorously shaken by hand; 10 ml of the slurries were transferred to centrifuge tubes and then centrifuged at 10,000 rpm for 5 min to collect the soil pellets. The pellets were then stored at −20°C for molecular analysis. The supernatants were collected and stored at −20°C for inorganic nitrogen analysis. Inorganic nitrogen species (NH^+^_4_, NO^−^_3_, and NO^−^_2_) were extracted with 2 M KCl and analyzed using a continuous flow analyzer (SA1000, Skalar, Netherlands).

### Soil DNA extraction and quantitative polymerase chain reaction

DNA was extracted from approximately 0.5 g of soil pellet following the method of Griffiths et al. (2000) with slight modifications following a bead-beating step, which was performed in triplicate. The quality and quantity of the DNA was assessed using a NanoDrop spectrophotometer (NanoDrop Technologies, Wilmington, DE). Real-time quantitative PCR (qPCR) with three replicates for each sample was performed to determine the copy numbers of the *amoA* and *pmoA* genes using the primer sets Arch-amoAF/Arch-amoAR for AOA (Francis et al., 2005), amoA-1F/amoA-2R-GG for AOB (Rotthauwe et al., 1997) and A189f/mb661r for MOB (Costello and Lidstrom, 1999) with a CFX96 Optical Real-Time Detection System (Bio-Rad Laboratories, Hercules, CA). The qPCR standard was generated using plasmid DNA from representative clones containing the bacterial or archaeal *amoA* gene or bacterial *pmoA* gene. A dilution series of a standard template across six orders of magnitude (3.12 × 10^2^ to 3.12 × 10^8^ for AOB, 1.56 × 10^2^ to 1.56 × 10^8^ for AOA and 1.82 × 10^2^ to 1.82 × 10^8^ for MOB) per assay was used to optimize the qPCR conditions. The blank was always run with water as the template instead of the soil DNA extract. The 20 μl reaction mixture contained 10.0 μl of SYBR Premix Ex Taq (TaKaRa Biotech, Dalian, China), 0.25 μM of each primer, and 2 μl of DNA template. The PCR conditions used for the archaeal and bacterial *amoA* genes were the same as previously described (Jia and Conrad, 2009). For the *pmoA* gene amplification, the PCR conditions were as follows: initial denaturation at 95°C for 30 s; 40 cycles consisting of denaturation at 95°C for 10 s, primer annealing at 55°C for 30 s and elongation at 72°C for 30 s. PCR amplification efficiencies of 101.9% with a *R*^2^ value of 0.998, 99.2% with a *R*^2^ value of 0.990 and 101.4% with a *R*^2^ value of 0.993 were obtained for the archaeal *amoA* gene, the bacterial *amoA* gene and the *pmoA* gene, respectively. The specific amplifications of *amoA* and *pmoA* were also determined using a melting curve analysis, which always resulted in a single peak.

### Polymerase chain reaction–denaturing gradient gel electrophoresis (DGGE)

For the DGGE analysis, PCR amplification of the archaeal and bacterial *amoA* gene was performed using the same primers as described above; however, the forward primer for the bacterial *amoA* was attached to a GC-clamp. The PCR reaction was performed in a 25 μl volume containing 2.5 μl 10 × PCR buffer, 0.25 μM of each primer, 200 μM (each) deoxyribonucleoside triphosphate, 1.5 U of Taq DNA polymerase, and 1 μl of soil DNA. The PCR was performed in a Thermal Cycler Dice (Takara Bio, Shiga, Japan), as previously described for the AOA (Francis et al., 2005) and AOB (Nicolaisen and Ramsing, 2002). The PCR products were run in a 1.5% agarose gel to determine their specificity and were spectrophotometrically measured to determine their concentrations.

Approximately 150 ng of PCR amplicons from each sample was subjected to DGGE analysis. For AOA, the PCR products were run in 6% acrylamide gels with a denaturing gradient of 20–50% (100% denaturant corresponds to 7 M urea and 40% deionized formamide). For AOB, an 8% gel with a 45–75% denaturing gradient was used. The gels were run in 1 × TAE at 75 V for 17 h and stained with SYBR Green I dye. The stained gels were imaged (Gel Doc system, Bio-Rad Laboratories, Hercules, CA), digitized, and processed (Gelcompar II, Applied Maths, Inc., Austin, TX).

### Sequencing and phylogenetic analysis

The distinct DGGE bands for the *amoA* genes of AOB (10 bands) and AOA (5 bands) among all the treatments were excised and re-amplified using the previously described PCR conditions. These PCR products were cloned using the pEasy-T1 cloning kit (TransGen Biotech Co., Beijing). The clones that contained the correct insert were selected and sequenced using an ABI 3730 XL DNA analyzer (Beijing Genomics Institute, Beijing, China). For MOB, the PCR products from the five treatments [original soil sample (day 0), 4th week Urea-0 (U-0), 4th week Urea-400 (U-400), 4th week AS-0 (AS-0), and 4th week AS-200 (AS-200)] were directly used for the cloning to construct a clone library. The cloning was performed following the same procedure as described above. At least 6–12 clones were randomly selected for each treatment. The sequences for the DGGE bands and the clones as well as their closest relatives obtained by BLAST analyses (http://blast.ncbi.nlm.nih.gov/Blast.cgi) were aligned using CLUSTAL X 1.83 (Thompson et al., 1997). Phylogenetic trees were constructed using the neighbor-joining method based on the Jukes–Cantor correction MEGA (Molecular Evolutionary Genetics Analysis) version 4 (Tamura et al., 2007).

### Statistics

Spearman's correlation analyses were performed to assess the relationships among methane oxidation activity, nitrification activity and the abundance of the MOB (SPSS 11.5 package, SPSS, Chicago, IL). A One-Way ANOVA with Duncan's *post hoc* tests was performed to evaluate the differences within the datasets, with a *P* value of 0.05 selected for significance.

### Nucleotide sequence accession numbers

The sequences obtained in this study have been deposited in GenBank with the accession numbers JQ990075–JQ990084, affiliated with the AOB; JQ990070–JQ990074, affiliated with the AOA and JQ990085–JQ990125, affiliated with the MOB.

## Results

### CH_4_ oxidation activity

**Figure 1:**
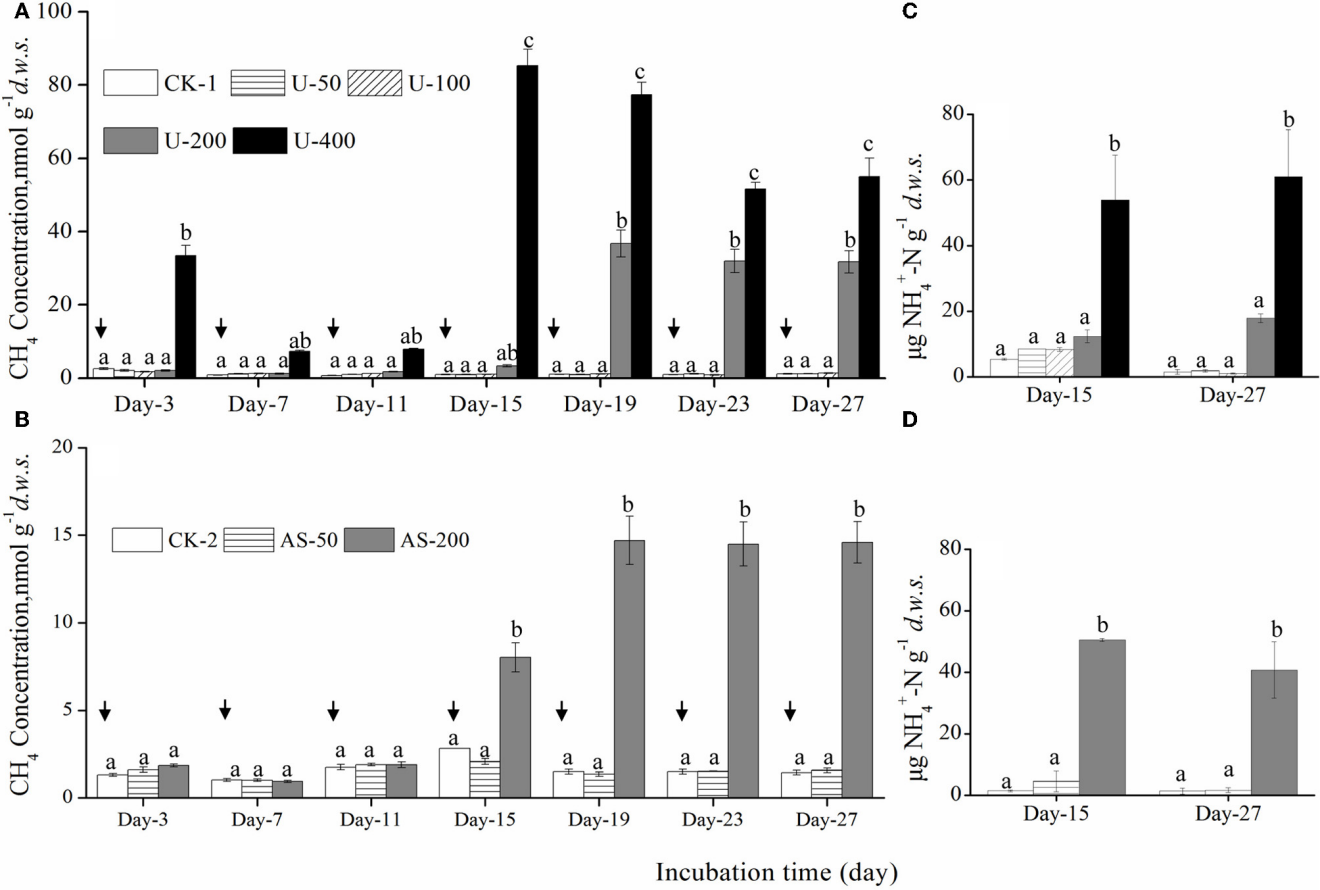
**Changes in the CH_4_ concentrations in the headspaces of soil microcosms amended with urea (A)** or ammonium sulfate **(B)** over an incubation course of 27 days as well as changes in the concentrations of NH^+^_4_-N in the soil microcosms **(C,D)**. The designations CK-1, U-50, U-100, U-200 and U-400 represent the soil microcosms that received no fertilization, 50, 100, 200, and 400 μg urea-N/*g d.w.s*., respectively. The designations CK-2, AS-50, and AS-200 represent the soil microcosms treated with no fertilization, 50 and 200 μg ammonium sulfate-N/*g d.w.s*., respectively. The arrows indicate the repeated addition of CH_4_, i.e., an initial concentration of ~ 5,000 ppm CH_4_ in the headspace was re-established after an incubation period of approximately three days. Error bars represent the standard deviation of the duplicate microcosms, and the same letter above the columns refers to no statistically significant difference among the treatments (*P* > 0.05).

The initial CH_4_ concentration in the headspace was approximately 2.5 μmol g^−1^ *d.w.s*., and methane oxidation was influenced by nitrogenous fertilizers during the microcosm incubations (Figure 1). After 27 days of incubation, there were no statistically significant differences in the CH_4_ concentrations among the soil microcosms amended with 0, 50, and 100 μg urea N/g.*d.w.s*. (1.14, 1.20, and 1.32 nmol/g.*d.w.s*., respectively), but significantly higher CH_4_ concentrations were observed in the headspaces of the soil microcosms containing 200 and 400 μg urea N/g.*d.w.s* (31.7 and 55.0 nmol/g.*d.w.s*., respectively; Figure 1A). The methane concentrations were 1.57 and 1.45 nmol/g.*d.w.s*. in the microcosms treated with ammonium sulfate at concentrations of 0 and 50 μg N/g.*d.w.s*., respectively (Figure 1B). A significantly higher CH_4_ concentration of 14.6 nmol/g.*d.w.s*. was observed in the microcosms when treated with 200 μg (NH_4_)_2_SO_4_-N/g.*d.w.s*. It is interesting to note that the ammonium concentrations were remarkably higher in the soil microcosms where CH_4_ oxidation was apparently inhibited by U-400 and AS-200 (Figures 1C,D). For example, 61 μg NH^+^_4_-N g^−1^*d.w.s*. was observed in the soil microcosms treated with 400 μg urea N/g.*d.w.s*., which is compared to 1.5 μg NH^+^_4_-N g^−1^*d.w.s* in the soil microcosms that received no nitrogenous fertilizers.

### Nitrification activity

**Figure 2:**
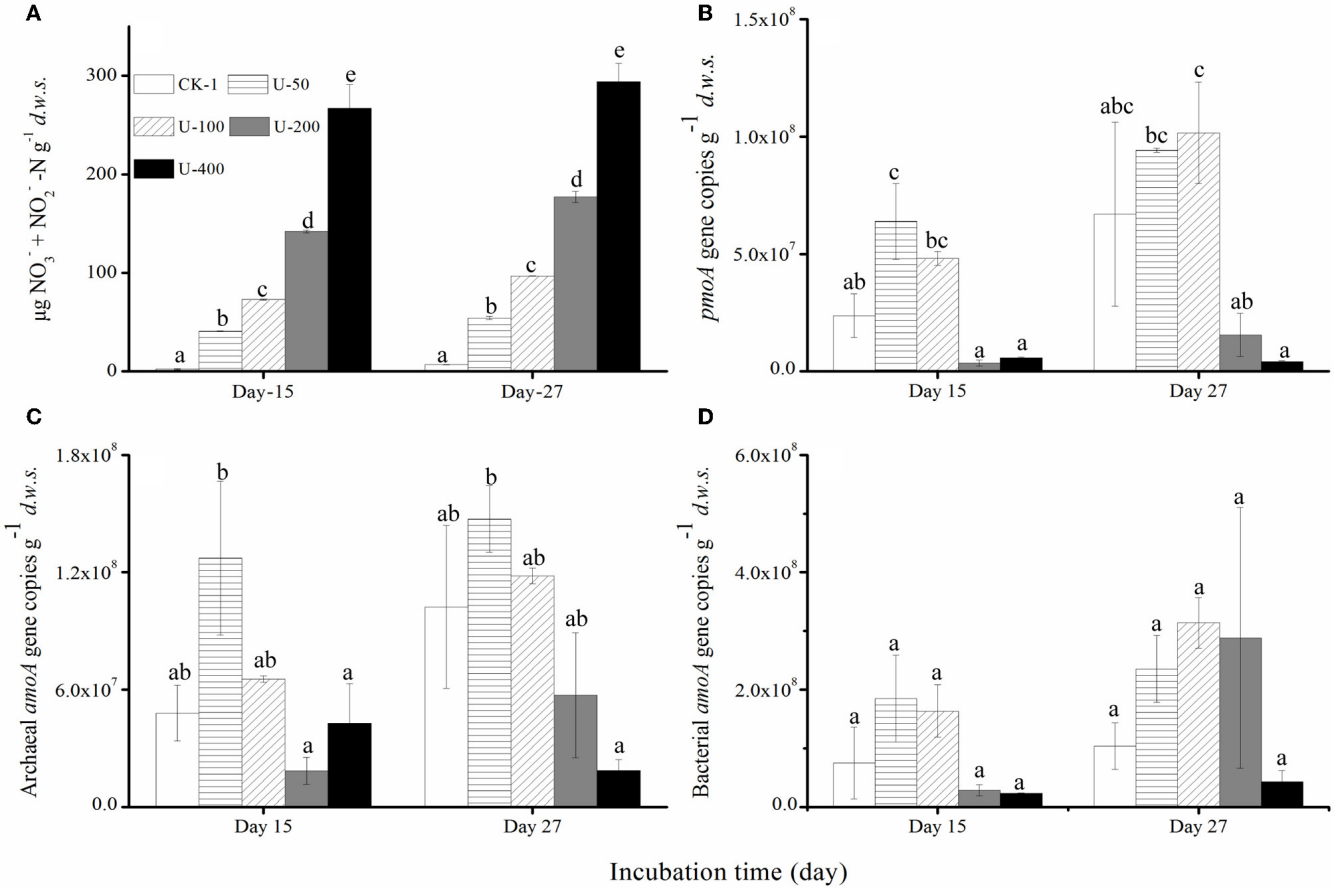
**Changes in the concentrations of NO^−^_3_ and NO^−^_3_-N (A)** the abundances of the *pmoA* genes of the methane-oxidizing bacteria **(B)** and the *amoA* genes of the *Archaea*** (C)** and *Bacteria* **(D)** in the soil microcosms amended with urea. The designations are the same as in Figure 1. The error bars represent the standard deviation of the duplicate microcosms, and the same letter above the columns refers to no statistically significant difference among the treatments (*P* > 0.05).

**Figure 3:**
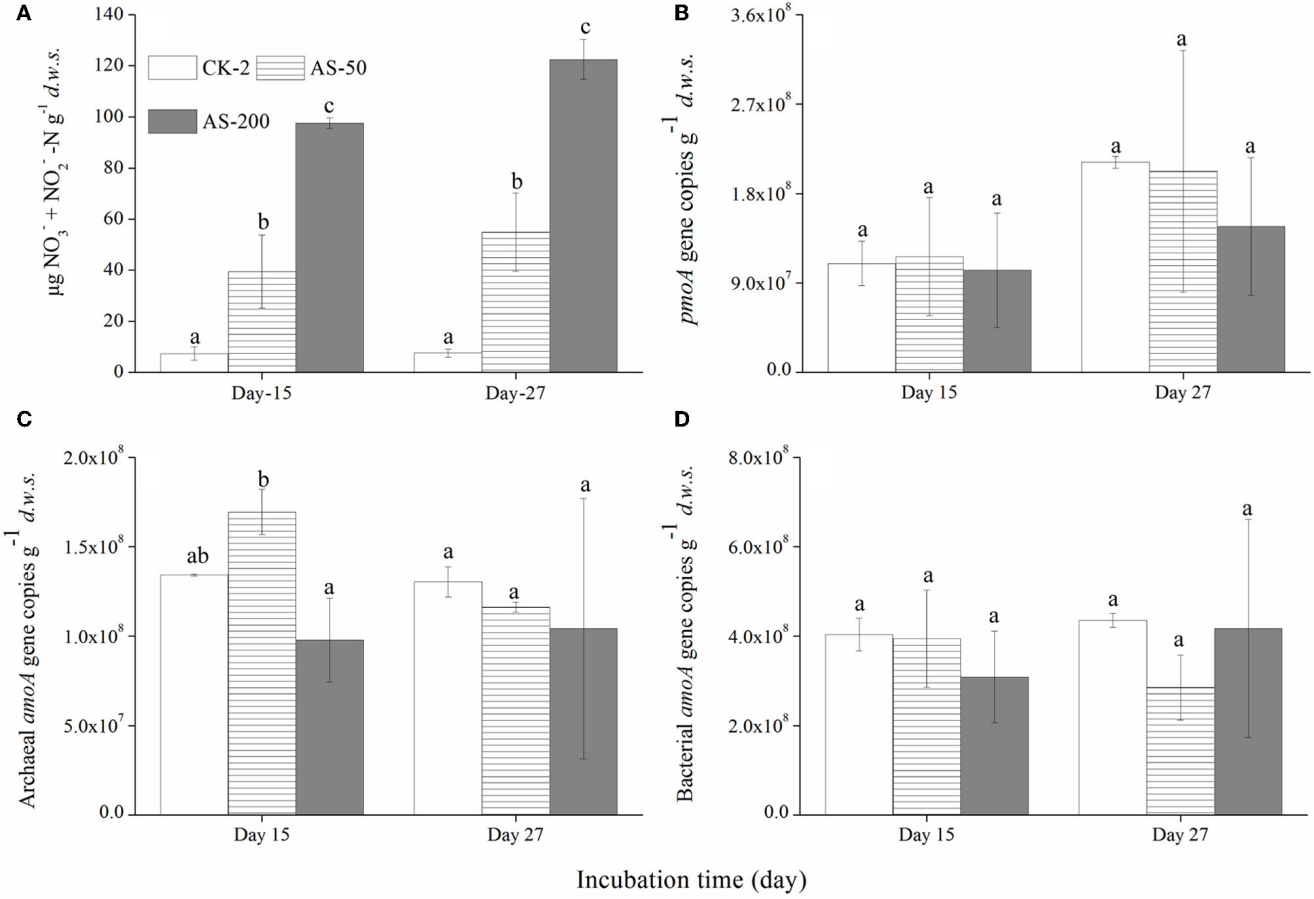
**Changes in the concentration of NO^−^_3_ and NO^−^_3_-N (A)** the abundances of the *pmoA* genes of the methane-oxidizing bacteria **(B)** and the *amoA* genes of the *Archaea* **(C)** and *Bacteria* **(D)** in the soil microcosms amended with ammonium sulfate. The designations are the same as in Figure 1. The error bars represent the standard deviation of the duplicate microcosms, and the same letter above the columns refers to no statistically significant difference among the treatments (*P* > 0.05).

Regardless of the urea and (NH_4_)_2_SO_4_ treatments, there was a strong nitrification activity in the presence of CH_4_. The production of nitrate and nitrite in the soil microcosms was positively related to the amounts of urea or (NH_4_)_2_SO_4_ added (Figures 2A and 3A) while the native soil contained only 1.54 μg (nitrate + nitrite) N g^−1^ *d.w.s*. All the microcosms treated with urea and (NH_4_)_2_SO_4_ displayed significant productions of soil nitrite and nitrate, demonstrating a strong nitrification activity over the course of the 27-day incubation. After 27 days of incubation, the soil nitrate and nitrite concentrations reached 294 μg N/g.*d.w.s*. and 123 μg N/g.*d.w.s*. in the microcosms amended with 400 μg urea-N/g.*d.w.s*. and 200 μg (NH_4_)_2_SO_4_-N/g.*d.w.s*., respectively (Figures 2A and 3A). As for the ammonium concentrations, 7.45 μg N/g.*d.w.s*. was observed in the native soil, and the consumed ammonium was recovered in an almost stoichiometric amount to the nitrate and nitrite produced in the soil microcosm after 15 or 27 days of incubation. Similar findings were observed in the soil microcosms amended with ammonium sulfate.

### Abundance of MOB, AOB, and AOA communities

The abundances of MOB, AOB, and AOA were determined using a qPCR targeting the *pmoA* and *amoA* genes (Figures 2B–D and 3B–D). The *pmoA* gene copy number varied significantly in the soil microcosms with different nitrogenous substrates. For the native soil, the copy number of the *pmoA* genes was 1.37 × 10^7^ g^−1^ *d.w.s*. After 27 days of incubation, the *pmoA* gene copy numbers were 9.42 × 10^7^ and 1.02 × 10^8^ in the soil microcosms treated with 50 and 100 μg urea N/g.*d.w.s*, respectively. In the microcosms containing 200 and 400 μg urea N/g.*d.w.s*., copy numbers of 1.56 × 10^7^ and 4.27 × 10^6^ were observed as well as 6.70 × 10^7^ *pmoA* genes within the control microcosms receiving no urea. Similarly, the *pmoA* gene copy numbers decreased slightly in the soil microcosms containing 50 and 200 μg (NH_4_)_2_SO_4_-N/g.*d.w.s* compared to the control microcosms receiving no (NH_4_)_2_SO_4_-N. After 27 days of incubation, the copy numbers of the *pmoA* genes were 2.1 × 10^8^, 2.0 × 10^8^ and 1.5 × 10^8^ in the soil microcosms treated with 0, 50, and 200 μg (NH_4_)_2_SO_4_-N/g.*d.w.s*., respectively.

The abundance of AOB and AOA *amoA* genes also varied within the soil microcosms amended with the different nitrogenous fertilizers. For the native soil, the bacterial and archaeal gene copy numbers were 1.34 × 10^8^ and 1.12 × 10^8^, respectively. After 27 days of incubation, the bacterial *amoA* gene copy number increased with the application of 50, 100, and 200 μg urea N/g.*d.w.s*, as compared to the control treatment, while a decrease in the copy number was observed in microcosms treated with 400 μg urea N/g.*d.w.s*. Similar results were observed for the copy numbers of the archaeal *amoA* genes, and a lower abundance of 1.87 × 10^7^ was found in the soil microcosms that received the highest addition of N (400 μg urea N/g.*d.w.s*.). As for the (NH_4_)_2_SO_4_ treatment, the abundances of MOB, AOA and AOB appeared to remain constant over the course of the incubation (Figures 3B–D)

### Relating methane and ammonia oxidations with functional gene abundances

**Figure A1:**
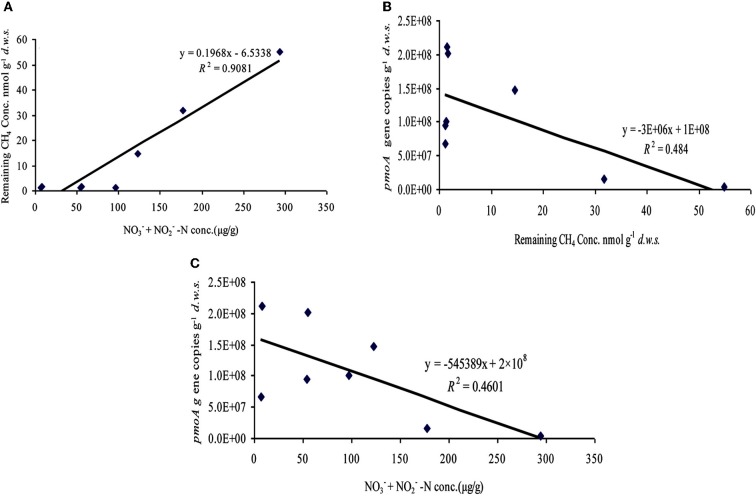
**Relationships among the concentration of CH_4_ remained in the headspace, the amount of NO^−^_2_ and NO^−^_3_-N and the copy number of *pmoA* genes in the soil microcosms amended with urea and ammonium sulfate after 27 days of incubation.** The black line is the linear regression line between two parameters. The *P* values for panel **(A)** 0.001, **(B)** 0.055, and **(C)** 0.064.

Regression analysis among the concentration of CH_4_ remained in the headspace, the amount of NO^−^_2_ and NO^−^_3_-N and the copy number of *pmoA* genes in the soil microcosms amended with urea and ammonium sulfate after 27 days of incubation were performed (Figure A1). Despite not being statistically significant (*R*^2^ = 0.484), CH_4_ oxidation was positively correlated with *pmoA* gene copy number. For instance, soil microcosms containing 200 and 400 μg urea N/g.*d.w.s* displayed a decreased *pmoA* gene copy number with lower CH_4_ oxidation rates after 27 days of incubation, as compared to the control treatment. Similar results were obtained for ammonium sulfate-amended treatment Interestingly, CH_4_ oxidation activity was significantly negatively correlated with the concentrations of NO^−^_2_ plus NO^−^_3_-N in the presence of urea and/or ammonium sulfate (ρ = −0.838), and the copy number of *pmoA* genes was negatively correlated with the concentration of NO^−^_2_ plus NO^−^_3_-N (ρ = −0.485). Moreover, the ratio of AOA to AOB was significantly positively correlated with CH_4_ oxidation activity (ρ = 0.57) but negatively correlated with the concentration of NO^−^_2_ plus NO^−^_3_-N (ρ = −0.48).

### Community compositions of MOB, AOB, and AOA

**Figure 4:**
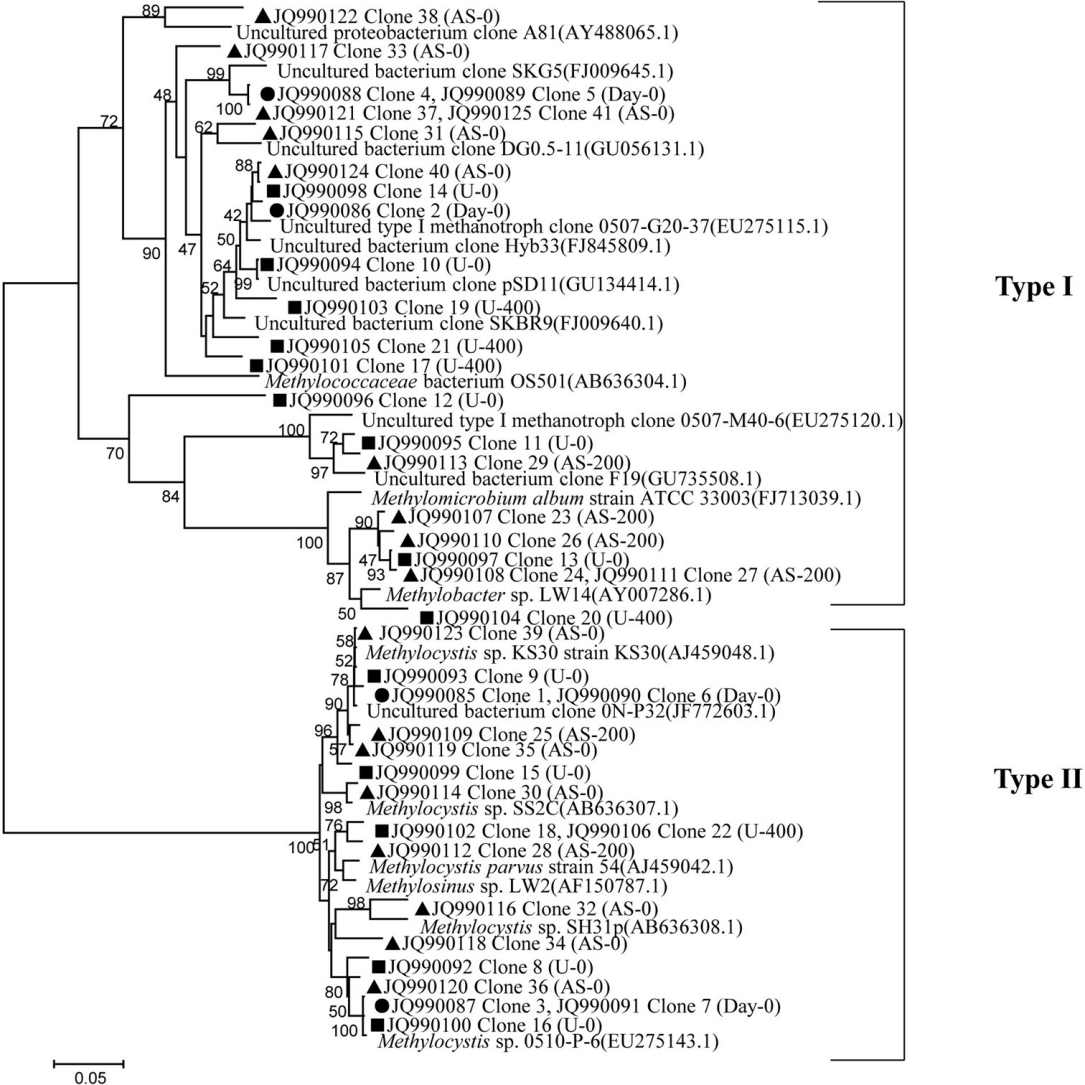
**Neighbor-joining tree showing the relationships of the *pmoA* genes retrieved from the clone library in this study to those in GenBank.** The scale bar indicates 5 changes per 100 nucleotide positions. Bootstrap values (>40%) are indicated at the branch points. • represents the native soil (day 0), and ▲ and ■ represent the ammonium sulfate-and urea-amended soil microcosms, respectively.

Clone library of *pmoA* genes was constructed, and phylogenetic analyses indicated contrasting changes of type I and II MOBs among the treatments (Figure 4). In total, 23 out of 41 sequences (up to 56%) were classified as type I MOB, and the remaining 18 sequences were related to type II MOB. For the native soil sample, the ratio of type I to type II MOB was 0.75. After a 27-day incubation period, the ratios of type I to type II MOB were 1.22, 2.03, 1.00, and 2.45 for the U-0, U-400, AS-0, and AS-200 treatments, respectively. The type I MOB were affiliated with the *Methylobacter*, *Methylomicrobium*, *Methylococcus* and uncultured methanotrophic clones, whereas the type II clones were phylogenetically related to the *Methylocystis* and *Methylosinus* genera and uncultured clones.

**Figure 5:**
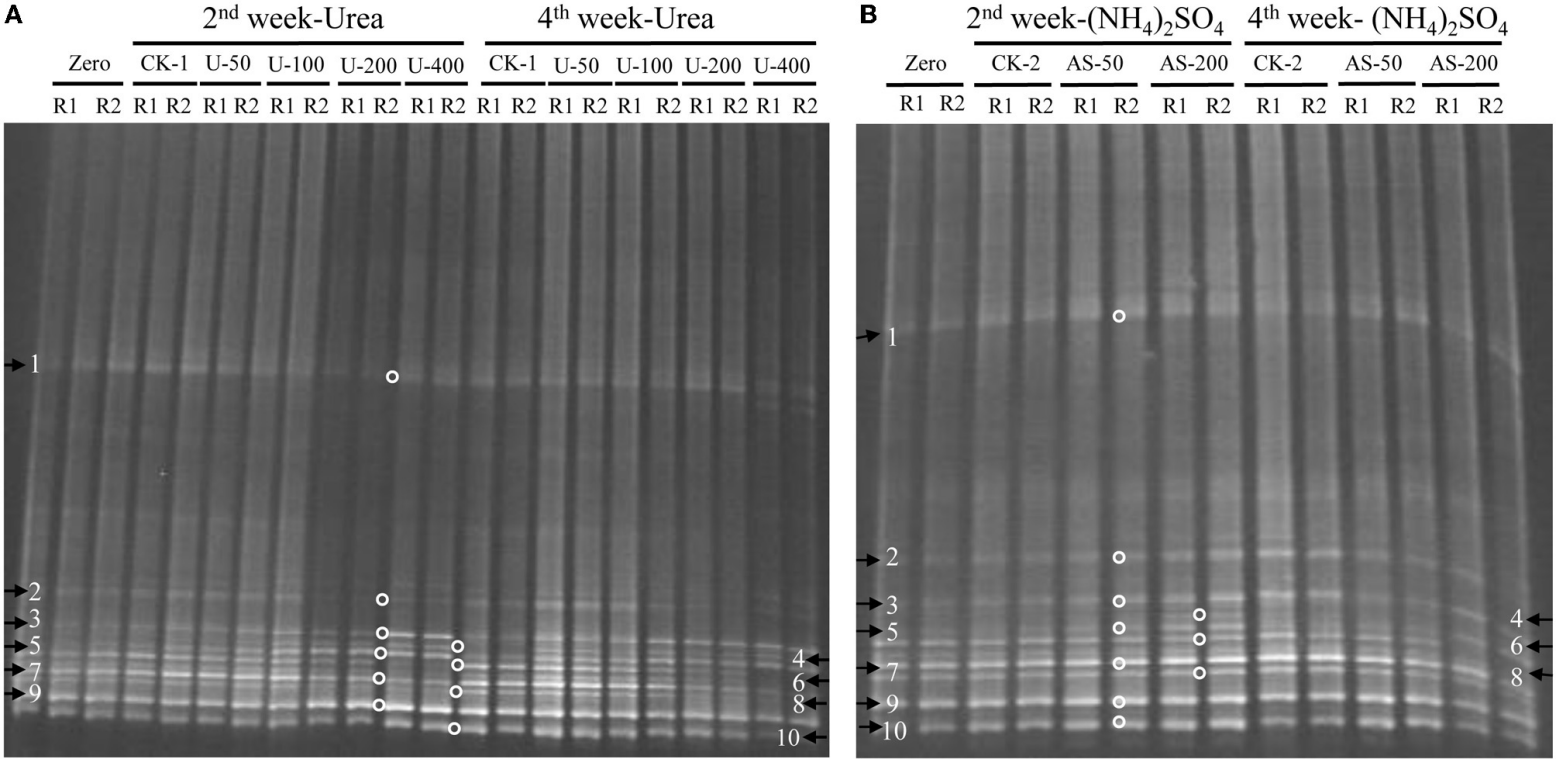
**DGGE fingerprints of the bacterial *amoA* gene in the soil microcosms amended with urea **(A)** and ammonium sulfate **(B)**.** The arrows indicate DGGE bands excised for sequencing. Zero represents the original soil sample, and all other designations are the same as in Figure 1. R1 and R2 represent duplicate microcosms.

**Figure 6:**
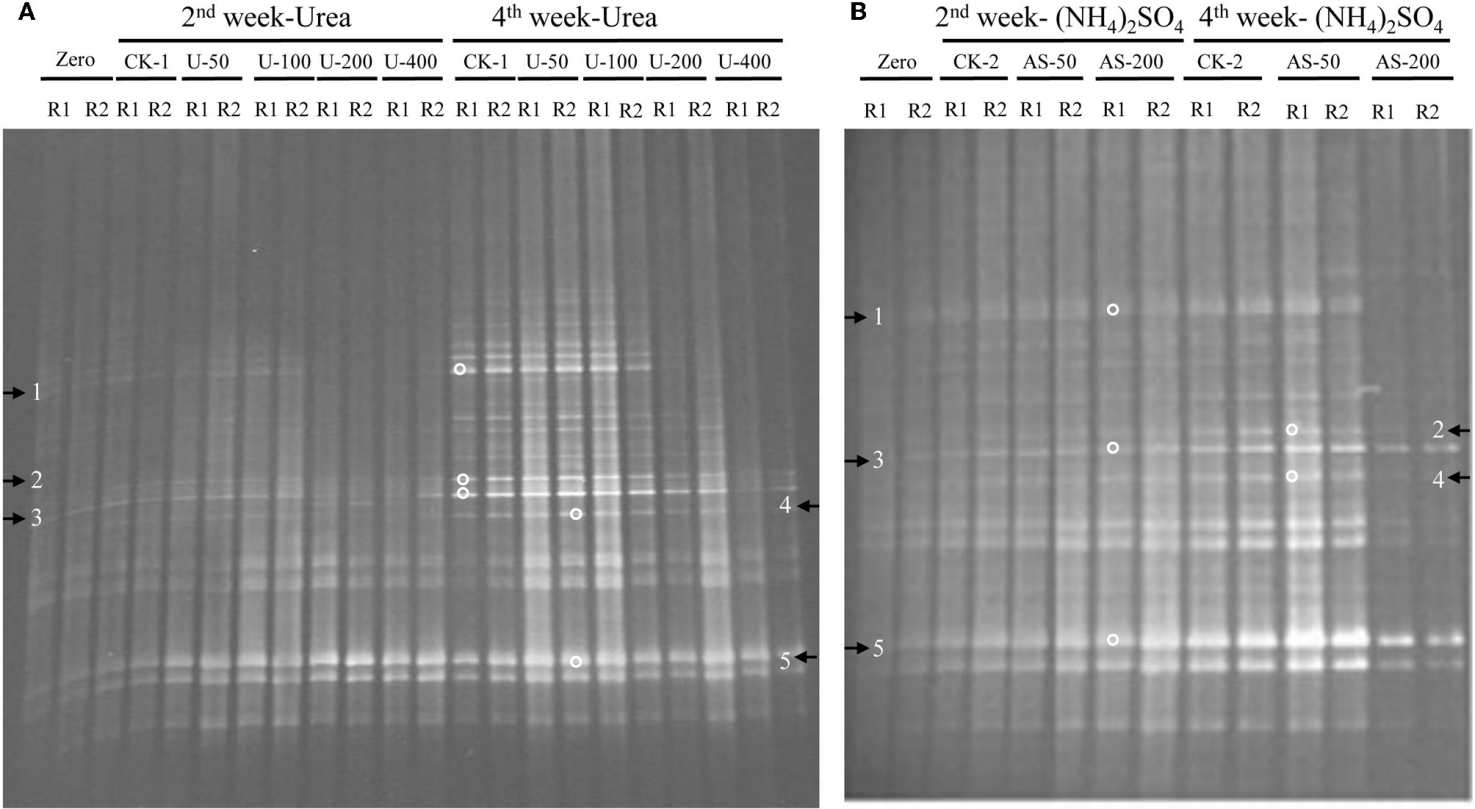
**DGGE fingerprints of the archaeal *amoA* gene in the soil microcosms amended with urea **(A)** and ammonium sulfate **(B)**.** The arrows indicate DGGE bands excised for sequencing. Zero represents the original soil sample, and all other designations are the same as those in Figure 1. R1 and R2 represent duplicate microcosms.

**Figure 7:**
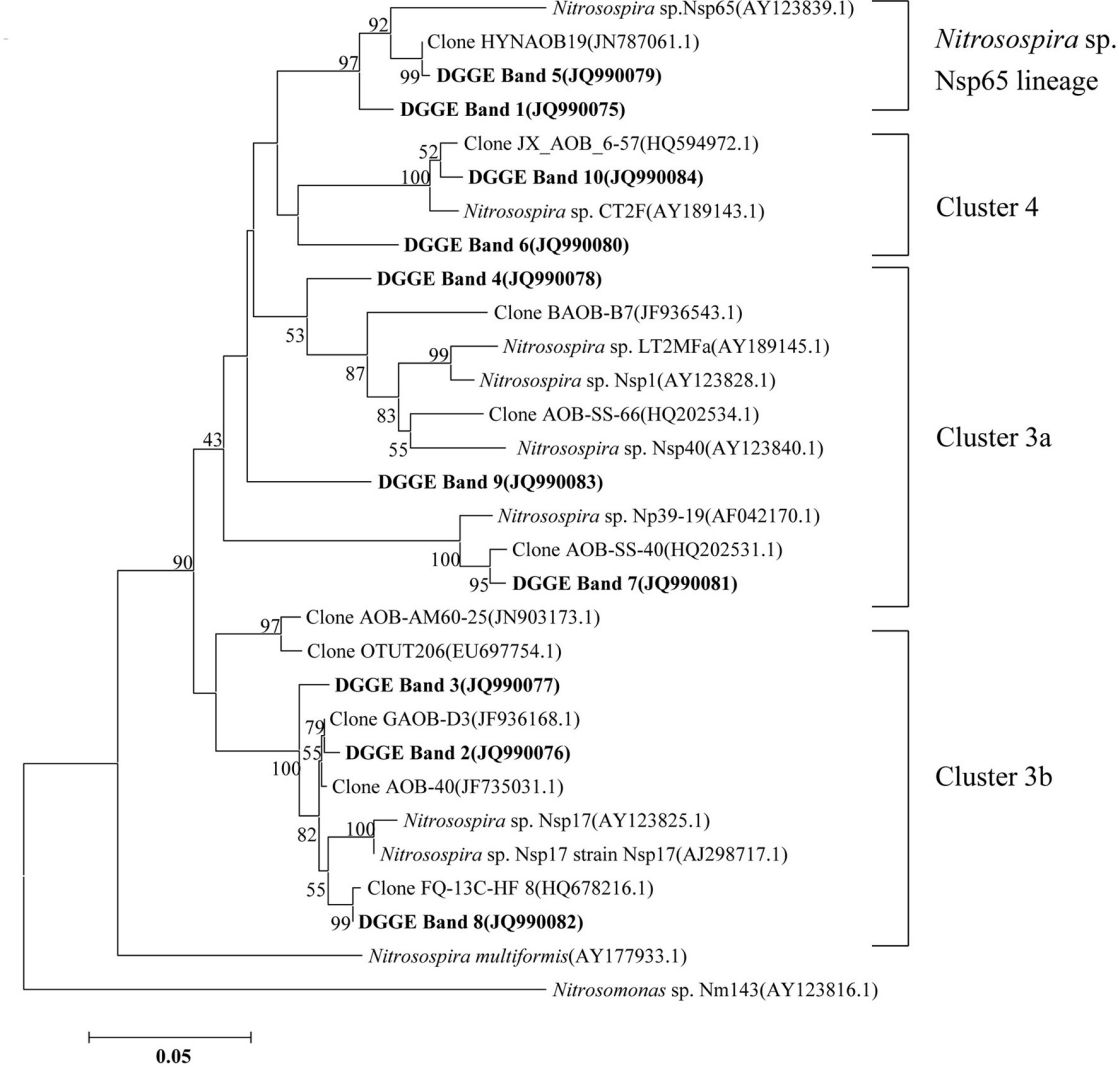
**Neighbor-joining tree showing the relationships of the bacterial *amoA* genes retrieved from the DGGE bands (bold) in this study to those in the GenBank.** The scale bar indicates 5 changes per 100 nucleotide acid positions. Bootstrap values (>40%) are indicated at branch points.

**Figure 8:**
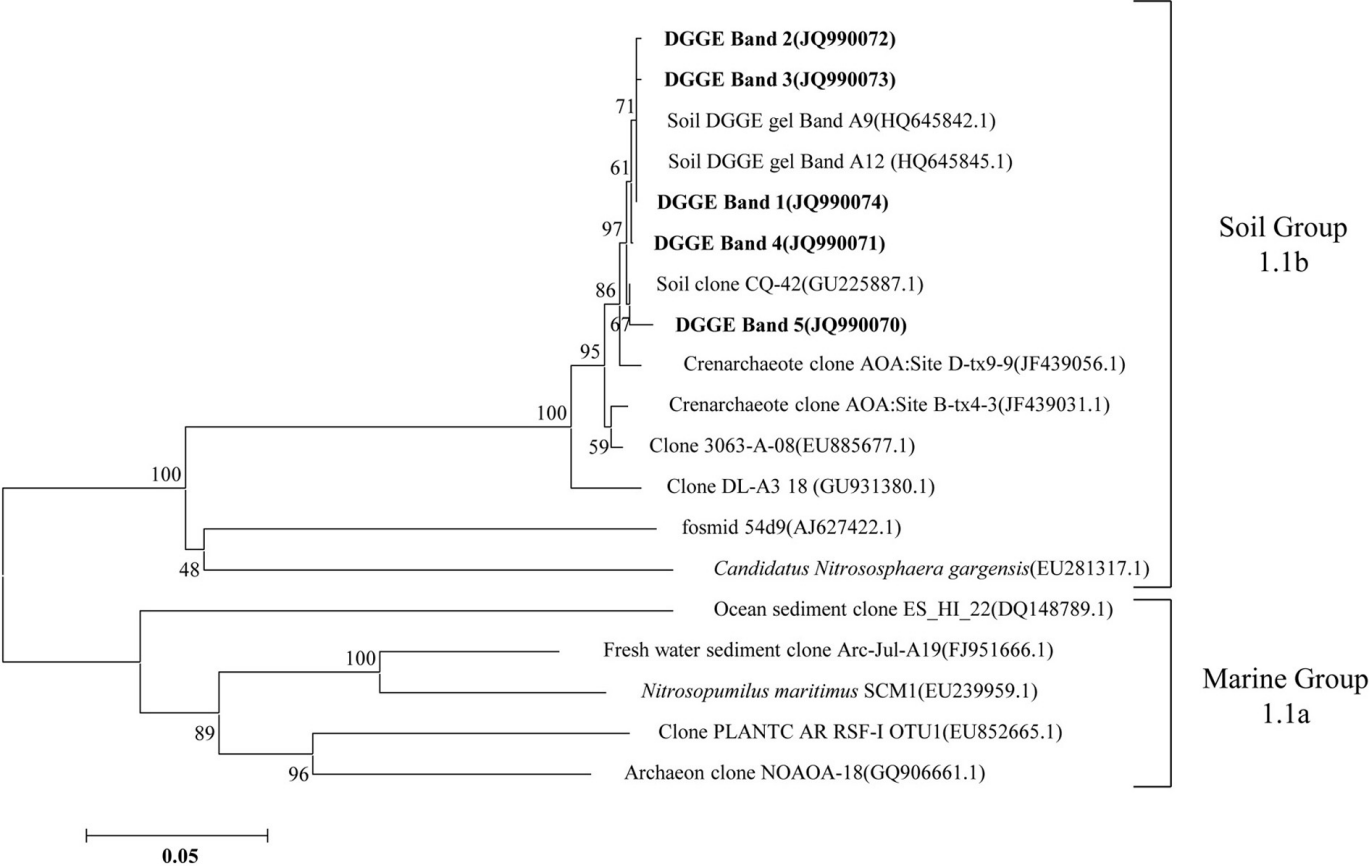
**Neighbor-joining tree showing the relationships of the archaeal *amoA* genes retrieved from the DGGE bands (bold) in this study to those in the GenBank.** The scale bar indicates 5 changes per 100 nucleotide acid positions. Bootstrap values (>40%) are indicated at branch points.

The compositions of the AOB and AOA communities were revealed by DGGE fingerprinting analyses of the bacterial and archaeal *amoA* genes in duplicate microcosms for the urea and (NH_4_)_2_SO_4_ treatments (Figures 5 and 6). Distinctly different DGGE fingerprints for the bacterial *amoA* genes were observed among the control treatment and the soil microcosms amended with different levels of nitrogenous fertilizers. Dominant AOB DGGE bands (1–10) were sequenced for phylogenetic analysis (Figure 7). Six DGGE bands (2–4 and 7–9) were affiliated with the *Nitrosospira* cluster 3 lineage, whereas bands 1 and 5 clustered with the taxonomically unclassified *Nitrosospira* sp. Nsp65 lineage. Additionally, DGGE bands 6 and 10 were related to *Nitrosospira* cluster 4. Comparisons of the DGGE patterns among the different treatments indicated that the relative intensities of DGGE bands 6 and 8 decreased upon the addition of higher levels of urea, whereas band 6 disappeared in the soil amended with 200 μg (NH_4_)_2_SO_4_ N/g.*d.w.s*. As for the AOA, 5 DGGE bands for the archaeal *amoA* gene were excised for sequencing (Figure 8). DGGE band 1 was either absent or the intensity of its band was lower in the soil microcosms treated with heavy fertilizations. Phylogenetic analyses indicated that all of the AOA sequences fall well within the soil group I.1b lineage.

## Discussion

### Methane oxidation activity

The results of this study have revealed that CH_4_ oxidation activity in a paddy soil might be affected by nitrogenous fertilizations. CH_4_ oxidation was not significantly different among the microcosm treated with 0, 50 and 100 μg urea N/g.*d.w.s*. Similar results were observed for 0 and 50 μg (NH_4_)_2_SO_4_-N/g.*d.w.s*., indicating that the ammonium fertilizer had no significant effect on CH_4_ oxidation. These observations are largely consistent with previous findings (Dunfield et al., 1995; Delgado and Mosier, 1996; Dan et al., 2001). However, 200 and 400 μg urea N/g.*d.w.s* appeared to inhibit CH_4_ oxidation activity compared to other treatments. Interestingly, 200 μg (NH_4_)_2_SO_4_-N/g.*d.w.s*. also inhibited CH_4_ oxidation. These findings are in agreement with those reported by (Steudler et al., 1989; King and Schnell, 1994; Bosse et al., 1993; Gulledge et al., 1997; Van der Nat et al., 1997). The main mechanism by which nitrogenous fertilizers inhibits CH_4_ oxidation is thought to be ammonia, which competes with CH_4_ for the methane monooxygenases in MOB. Even though the affinity of MMO for CH_4_ is 600–1300-fold higher than its affinity for ammonia, excessively high concentrations of ammonium are known to substantially inhibit CH_4_ oxidation (Be'dard and Knowles, 1989). The results of this study support this mechanism because the inhibition of CH_4_ oxidation appeared to be intensified with higher concentrations of ammonium.

In this study, ammonium-based fertilizers displayed either an inhibitory effect or no effect on CH_4_ oxidation, which was dependent on the concentration of the applied fertilizers. To the contrary, Bodelier and co-workers (2000a,b) have elegantly demonstrated a stimulation of CH_4_ oxidation by ammonium fertilization in paddy soils. This observation might result from the absence of inorganic nitrogen, which would lead to an inactive and most likely non-growing methanotrophic community. Thus, the addition of ammonium-based fertilizers would relieve the nitrogen-limiting conditions and stimulate CH_4_ oxidation activity. It is likely that the soil used in this study was not constrained by the availability of N to support microbial growth. Therefore, the growth of methanotrophic communities was not restricted by the nitrogenous substrate, and no stimulation of CH_4_ oxidation activity was observed upon the addition of nitrogenous fertilizations. In addition to the soil's nitrogen status, our results also indicate a strong correlation between the concentration of the applied ammonium fertilizers and CH_4_ oxidation activity. Up to a certain concentration, ammonium-based fertilizers had no effect on CH_4_ oxidation; however, under higher concentrations, there was an inhibitory effect on the CH_4_ oxidation rate. CH_4_ oxidation in the paddy soils may have been inhibited when the concentration of urea/(NH_4_)_2_SO_4_ reached 200 μg N/g.*d.w.s* or above.

### Nitrification activity

The nitrification activity responded positively to nitrogenous fertilizations. All the urea- and (NH_4_)_2_SO_4_-treated microcosms displayed gradual increased productions of nitrate and nitrite over the incubation period upon the addition of nitrogenous fertilizers. Similar results have been reported by Avrahami et al. (2002) and Verhamme et al. (2011), that is, nitrification activity increased with increasing concentrations of ammonium in soil microcosms. Although a negative correlation between nitrification activity and CH_4_ oxidation activity was observed, it is interesting to note that the presence of CH_4_ appeared to have no adverse effect on nitrification activity in this study. We speculate that the stimulated nitrification activity might have led to a soil pH decline, as reported previously (Jia and Conrad, 2009). The acidification of the soil microcosms could have further inhibited CH_4_ oxidation activity by suppressing the growth of MOB in soil microcosms amended with higher concentrations of the nitrogenous fertilizers. In addition, the elevated concentrations of soil nitrate might have had an adverse effect on CH_4_ oxidation. Reay and Nedwell (2004) and Xu and Inubushi (2004) have shown a negative correlation between nitrate concentrations in soils and CH_4_ oxidation rates in coniferous and deciduous forest soils. However, the mechanism has yet to be determined. Several studies have suggested that cations associated with nitrate rather than nitrate itself are the main factors producing the inhibitory effect, but contrasting results have also been reported (Wang and Ineson, 2003). A comprehensive investigation of the microorganisms involved in nitrogen turnover, such as AOA and AOB, would be helpful in better understanding the interactions between ammonia oxidation and CH_4_ consumption in paddy soil.

### Abundances of MOB, AOB, and AOA gene copy number

Soil microcosms amended with 50 and 100 μg urea N/g.*d.w.s*. displayed an increased abundance of *pmoA* gene copies while a low abundance was observed in microcosms treated with 200 and 400 μg urea N/g.*d.w.s*. after 27 days of incubation, as compared to the control treatment. The *pmoA* gene copy number was higher in 0 and 100 μg ammonium sulfate N/g.*d.w.s* amended treatments compared to the 200 μg ammonium sulfate N/g.*d.w.s* amended microcosms, where the CH_4_ oxidation rate was higher in the 0 and 50 μg ammonium sulfate N/g.*d.w.s* than that of the 200 μg ammonium sulfate N/g.*d.w.s* amended microcosm. This result suggests that this suppressed CH_4_ oxidation activity was most likely attributable to the low abundance of methanotrophic communities in the soil microcosms that received heavy fertilizations.

Despite the fact that an apparently low abundance was measured in soil microcosms treated with 400 μg urea N/g.*d.w.s*. after a 27-day incubation, the bacterial *amoA* gene copy number was not significantly different among the microcosms treated with different levels of nitrogenous fertilizers. Similar results were obtained for the (NH_4_)_2_SO_4_ treatments. As for the AOA, the archaeal *amoA* gene copy numbers remained largely unchanged irrespective of the urea and ammonium sulfate–N addition levels. However, the treatment that received the highest N fertilizations of 400 μg urea N/g.*d.w.s*. had relatively lower *amoA* gene copy numbers. This finding is in agreement with previous results using a German agricultural soil (Jia and Conrad, 2009), semi-arid and temperate grassland in China (Shen et al., 2011), and a grazed grassland soil treated with high doses of urine-N input in New Zealand (Di et al., 2010).

### Community composition of MOB, AOB, and AOA

Distinct differences were found between the U-0 and U-400 treatments after four weeks of incubation, indicating that the MOB community was substantially altered upon the application of a higher amount of N-fertilizer. The soil microcosm amended with ammonium sulfate also displayed relative changes of the MOB community compared with the control treatment. Our results indicate that the type I MOB related to the *Methylobacter, Methylomicrobium*, and *Methylococcus* genera were less abundant than the type II MOB in the native soils studied. However, a relative increase of the type I MOB was observed in the soil microcosms amended with nitrogenous fertilizers while the type II MOB appeared to be inhibited. This observation is consistent with findings from forest and rice field soils (Mohanty et al., 2006). This result could be an effect of a competition for N between the type I and type II MOB during the incubations. With respect to the effects of the nitrogenous fertilizers on CH_4_ consumption, it appears that no consistent patterns can be generalized (Mohanty et al., 2006). Currently, the established inhibition mechanisms of CH_4_ oxidation by ammonium application are that the type I MOB benefit significantly more from the presence of inorganic nitrogen than the type II MOB, possibly because of the ability of type II MOB to fix molecular nitrogen (Graham et al., 1993; Bodelier et al., 2000b; Bodelier and Laanbroek, 2004). The rapid response of the type I MOB to N-addition may also be connected to nitrogen fixation, which is a capability that is widespread among MOB (Auman et al., 2001).

The effect of ammonium fertilizers on CH_4_ oxidation (stimulation or inhibition) obviously depends on the community's composition and, hence, on the biodiversity of the MOB present. Generally, it is assumed that CH_4_ consumption in a soil or sediment with a predominant type I MOB will not be affected by fertilizer application while CH_4_ uptake by a soil or sediment containing a predominant type II will be inhibited (Mohanty et al., 2006). Moreover, differentiation can also be expected within type I and II representatives. This scenario was clearly evident in the dominance of a specific type of MOB affiliated with *Methylocella* and *Methylocystis* in acid peat (Dedysh et al., 2001), a phylotype in association with *Methylocystis* and USCγ in periodically water-saturated gleyic soils (Knief et al., 2006) and *Methylomonas*-like MOBs in lake sediments (Auman and Lidstrom, 2002; Eller et al., 2005). In this study, the native soil was clearly dominated by type II MOB, which could have very likely been inhibited by higher levels of ammonium fertilizer.

The DGGE fingerprints of the bacterial *amoA* genes were altered by the addition of higher doses of nitrogenous fertilizers. A pairwise comparison of the DGGE patterns among the different treatments indicated that the relative intensities of DGGE bands 6 and 8 were significantly decreased within the soil microcosms containing higher levels of urea, whereas DGGE band 6 was absent in the soils that received 200 μg (NH_4_)_2_SO_4_-N/g.*d.w.s*. Phylogenetic analyses indicated that DGGE band 6 was most closely affiliated with the *Nitrosospira* cluster 4 group while DGGE band 8 clustered with the *Nistrosospira* cluster 3b group. We speculate that the AOB affiliated with DGGE bands 6 and 8 might not be the dominant ammonia oxidizers in the soils studied. It is very interesting to note that the dominant AOB DGGE bands were associated with *Nistrosospira* cluster 3 and the taxonomically unclassified *Nitrosospira* sp. Nsp65 lineage that could have been responsible for the stimulated nitrification activity observed in this study, which is consistent with the active ammonia oxidizers in an upland agricultural soil (Jia and Conrad, 2009). The interaction mechanisms between AOB and MOB remain unclear in a complex environment. For example, the contributions of MOB and AOB to ammonia oxidation and CH_4_ oxidation, respectively, and their possible interactions remain unresolved in complex environment (Bodelier, 2011). Furthermore, the discovery of AOA adds a new perspective to interactions between CH_4_ and ammonia oxidizers. AOA are very abundant and outnumber AOB in rice soils (Chen et al., 2008). However, it remains elusive as to whether CH_4_ oxidation could be affected by AOA in paddy soil and how AOA would compete with MOB for ammonium. Phylogenetic analyses demonstrated that archaeal ammonia oxidizers are dominated by AOA members within the soil Group I.1b. Although the DGGE fingerprints of the archaeal *amoA* genes displayed variations among the soil microcosms treated with different levels of nitrogenous fertilizers, such as DGGE band 1, this study provides no conclusive evidence that CH_4_ oxidation is linked to the AOA. Methodological developments to differentiate CH_4_ oxidation from ammonia oxidation under *in situ* conditions will be crucial for answering this question (Bodelier and Frenzel, 1999). It has very recently been shown that MOB can switch from CH_4_ oxidation to ammonia oxidation upon fertilizer addition by using stable C and N isotope probing (Acton and Baggs, 2011).

## Conclusion

Different levels of nitrogenous fertilizers can affect the CH_4_ oxidation activity as well as the abundance and composition of MOB. Inhibitory effects on CH_4_ oxidation were demonstrated in soil microcosms amended with 200 μg urea N/g.*d.w.s* and above after 27 days of incubation. Similar results were obtained for ammonium sulfate-amended soil microcosms. The community structure of MOB changed in the soil microcosms amended with different levels of nitrogenous fertilizers. The native MOB in the background soil were dominated by type II; however, the addition of ammonium stimulated type I MOB. In addition, our study indicated strong nitrification in soil microcosms amended with nitrogenous fertilizers. Strong nitrification might lead to a pH decline, which may affect the niche differentiation of MOB. The interaction mechanisms among AOA, AOB, and MOB will require further investigation.

### Conflict of interest statement

The authors declare that the research was conducted in the absence of any commercial or financial relationships that could be construed as a potential conflict of interest.

## References

[B1] Acton S. D. Baggs E. M. (2011). Interactions between N application rate, CH_4_ oxidation and N_2_O production in soil. Biogeochemistry 103, 15–26

[B2] Aronson E. L. Helliker B. R. (2010). Methane flux in non-wetland soils in response to nitrogen addition: a meta-analysis. Ecology 91, 3242–3251 21141185 10.1890/09-2185.1

[B3] Auman A. J. Lidstrom M. E. (2002). Analysis of sMMO-containing type I methanotrophs in Lake Washington sediment. Environ. Microbiol. 4, 517–524 10.1046/j.1462-2920.2002.00323.x 12220408

[B4] Auman A. J. Speake C. C. Lidstrom M. E. (2001). nifH sequences and nitrogen fixation in type I and type II methanotrophs. Appl. Environ. Microbiol. 67, 4009 10.1128/?AEM.67.9.4009-4016.2001 11525998 PMC93122

[B5] Avrahami S. Conrad R. Braker G. (2002). Effect of soil ammonium concentration on N_2_O release and on the community structure of ammonia oxidizers and denitrifiers. Appl. Environ. Microbiol. 68, 5685–5692 10.1128/AEM.68.11.5685-5692.2002 12406765 PMC129938

[B6] Be'dard C. Knowles R. (1989). Physiology, biochemistry, and specific inhibitors of CH_4_, NH^+^_4_, and CO oxidation by methanotrophs and nitrifiers. Microbiol. Rev. 53, 68–84 2496288 10.1128/mr.53.1.68-84.1989 PMC372717

[B7] Bodelier P. L. E. (2011). Interactions between nitrogenous fertilizers and methane cycling in wetland and upland soils. Curr. Opin. Environ. Sustainability. 3, 379–388

[B8] Bodelier P. L. E. Frenzel P. (1999). Contribution of methanotrophic and nitrifying bacteria to CH_4_ and NH^+^_4_ oxidation in the rhizosphere of rice plants as determined by new methods of discrimination. Appl. Environ. Microbiol. 65, 1826–1833 10223965 10.1128/aem.65.5.1826-1833.1999 PMC91262

[B9] Bodelier P. L. E. Hahn A. P. Arth I. R. Frenzel P. (2000a). Effects of ammonium based fertilisation on microbial processes involved in methane emission from soils planted with rice. Biogeochemistry 51, 225–257

[B11] Bodelier P. L. E. Roslev P. Henckel T. Frenzel P. (2000b). Stimulation by ammonium-based fertilizers of methane oxidation in soil around rice roots. Nature 403, 421–424 10.1038/35000193 10667792

[B10] Bodelier P. L. E. Laanbroek H. J. (2004). Nitrogen as a regulatory factor of methane oxidation in soils and sediments. FEMS Microbiol. Ecol. 47, 265–277 10.1016/S0168-6496(03)00304-0 19712315

[B12] Bosse U. Frenzel P. Conrad R. (1993). Inhibition of methane oxidation by ammonium in the surface layer of a littoral sediment. FEMS Microbiol. Ecol. 13, 123–134

[B13] Chen X. P. Zhu Y. G. Xia Y. Shen J. P. He J. Z. (2008). Ammonia-oxidizing archaea: important players in paddy rhizosphere soil? Environ. Microbiol. 10, 1978–1987 10.1111/j.1462-2920.2008.01613.x 18430011

[B14] Conrad R. (2004). “Methanogenic microbial communities associated with aquatic plants,” in Plant Surface Microbiology, eds Varma A. Abbott L. Werner D. Hampp R. (Berlin, Germany: Springer), 35–50

[B15] Costello A. M. Lidstrom M. E. (1999). Molecular characterization of functional and phylogenetic genes from natural populations of methanotrophs in lake sediments. Appl. Environ. Microbiol. 65, 5066–5074 10543824 10.1128/aem.65.11.5066-5074.1999 PMC91682

[B16] Crutzen P. J. (1995). “The role of methane in atmospheric chemistry and climate,” in Proceedings of the Eighth International Symposium on Ruminant Physiology, Ruminant Physiology: Digestion, Metabolism, Growth and Reproduction, eds Von Engelhardt W. Leonhard-Marek S. Breves G. Giesecke D. (Stuttgart, Germany: Ferdinand Enke Verlag), 291–316

[B17] Dan J. Krüger M. Frenzel P. Conrad R. (2001). Effect of a late season urea fertilization on methane emission from a rice field in Italy. Agric. Ecosyst. Environ. 69, 69–80

[B18] Dedysh S. N. Derakshani M. Liesack W. (2001). Detection and enumeration of methanotrophs in acidic Sphagnum peat by 16S rRNA fluorescence *in situ* hybridization, including the use of newly developed oligonucleotide probes for *Methylocella palustris* Appl. Environ. Microbiol. 67, 4850–4857 10.1128/AEM.67.10.4850-4857.2001 11571193 PMC93240

[B19] Delgado J. A. Mosier A. R. (1996). Mitigation alternatives to decrease nitrous oxides emissions and urea-nitrogen loss and their effect on methane flux. J. Environ. Qual. 25, 1105–1111

[B20] Di H. J. Cameron K. C. Shen J. P. Winefield C. S. O'Callaghan M. Bowatte S. He J. Z. (2010). Ammonia oxidizing bacteria and archaea grow under contrasting soil nitrogen conditions. FEMS Microbiol. Ecol. 72, 386–394 10.1111/j.1574-6941.2010.00861.x 20370827

[B21] Dunfield P. Knowles R. (1995). Kinetics of inhibition of methane oxidation by nitrate, nitrite and ammonium in a humisol. Appl. Environ. Microbiol. 61, 3129–3135 16535109 10.1128/aem.61.8.3129-3135.1995 PMC1388563

[B22] Dunfield P. F. Topp E. Archambault C. Knowles R. (1995). Effect of nitrogen fertilizers and moisture-content on CH_4_ and N_2_O fluxes in a humisol-measurements in the field and intact soil cores. Biogeochemistry 29, 199–222

[B23] Eller G. Deines P. Grey J. Richnow H.-H. Kruger M. (2005). Methane cycling in lake sediments and its influence on chironomid larval δ^13^C. FEMS Microbiol. Ecol. 54, 339–350 10.1016/j.femsec.2005.04.006 16332332

[B24] Francis C. A. Roberts K. J. Beman J. M. Santoro A. E. Oakley B. B. (2005). Ubiquity and diversity of ammonia-oxidizing archaea in water columns and sediments of the ocean. Proc. Natl. Acad. Sci. U.S.A. 102, 14683–14688 10.1073/pnas.0506625102 16186488 PMC1253578

[B25] Graham D. W. Chaudhary J. A. Hanson R. S. Ranold G. A. (1993). Factors affecting competition between type 1 and type 2 methanotrophs in two-organism, continuous-flow reactors. Microb. Ecol. 25, 1–17 10.1007/BF00182126 24189703

[B26] Griffiths R. I. Whiteley A. S. O'Donnell A. G. Bailey M. J. (2000). Rapid method for coextraction of DNA and RNA from natural environments for analysis of ribosomal DNA- and rRNA-based microbial community composition. Appl. Environ. Microbiol. 66, 5488–5491 10.1128/AEM.66.12.5488-5491.2000 11097934 PMC92488

[B27] Groot T. T. VanBodegom P. M. Harren F. J. M. Meijer H. A. J. (2003). Quantification of methane oxidation in the rice rhizosphere using ^13^C-labelled methane. Biogeochemistry 64, 355–372

[B28] Gulledge J. Doyle A. P. Schimel J. P. (1997). Different NH^+^_4_ inhibition patterns of soil CH_4_ –oxidizer populations across sites. Soil Biol. Biochem. 29, 13–21

[B29] Hanson R. S. Hanson T. E. (1996). Methanotrophic bacteria. Microbiol. Rev. 60, 439–471 8801441 10.1128/mr.60.2.439-471.1996 PMC239451

[B30] Houghton J. T. Ding Y. Griggs D. J. Noguer M. Van der Linden P. J. Xiaosu D. (eds.). (2001). Climate Change 2001: The Scientific Basis. Contribution of Working Group I to the Fourth Assessment Report of the Intergovernmental Panel on Climate Change. Cam-bridge: Cambridge University Press

[B31] IPCC (2007). “Climate change 2007: the physical science basis,” in Contribution of Working Group I to the Fourth Assessment Report of the Intergovernmental Panel on Climate Change, eds Solomon S. Qin D. Manning M. Chen Z. Marquis M. Averyt K. B. Tignor M. Miller H. L. (Cambridge, UK; New York, NY: Cambridge University Press), 996.

[B32] Jia Z. Conrad R. (2009). *Bacteria* rather than *Archaea* dominate microbial ammonia oxidation in an agricultural soil. Environ. Microbiol. 11, 1658–1671 10.1111/j.1462-2920.2009.01891.x 19236445

[B33] Khush G. S. (2005). What it will take to Feed 5.0 Billion Rice consumers in 2030. Plant Mol. Biol. 59, 1–6 10.1007/s11103-005-2159-5 16217597

[B34] King G. M. Schnell S. (1994). Effect of increasing atmospheric methane concentration on ammonium inhibition of soil methane consumption. Nature 370, 282–284

[B35] Knief C. Kolb S. Bodelier P. L. E. Lipski A. Dunfield P. (2006). The active methanotrophic community in hydromorphic soils changes in response to changing methane concentration. Environ. Microbiol. 8, 321–333 10.1111/j.1462-2920.2005.00898.x 16423018

[B36] Krüger M. Eller G. Conrad R. Frenzel P. (2002). Seasonal variation in pathways of CH_4_ oxidation in rice fields determined by stable carbon isotopes and specific inhibitors. Glob. Change Biol. 8, 265–280

[B37] Krüger M. Frenzel P. (2003). Effects of N-fertilization on CH_4_ oxidation and production, and consequences for CH_4_ emissions from microcosms and rice fields. Glob. Change Biol. 9, 773–784

[B38] Lelieveld J. Crutzen P. J. Dentener F. J. (1998). Changing concentrations, lifetime and climate forcing of atmospheric methane. Tellus 50B, 128–150

[B39] Liu D. Ding W. Jia Z. Cai Z. (2011). Relation between methanogenic archaea and methane production potential in selected natural wetland ecosystems across China. Biogeosciences 8, 329–338

[B40] Mohanty S. R. Bodelier P. L. E. Floris V. Conrad R. (2006). Differential effects of nitrogenous fertilizers on methane-consuming microbes in rice field and forest soils. Appl. Environ. Microbiol. 72, 1346–1354 10.1128/AEM.72.2.1346-1354.2006 16461686 PMC1392950

[B41] Nicolaisen M. H. Ramsing N. B. (2002). Denaturing gradient gel electrophoresis (DGGE) approaches to study the diversity of ammonia-oxidizing bacteria. J. Microbiol. Methods 50, 189–203 10.1016/S0167-7012(02)00026-X 11997169

[B42] Reay D. S. Nedwell D. B. (2004). Methane oxidation in temperate soils: effects of inorganic N. Soil Biol. Biochem. 36, 2059–2065

[B43] Rigby M. Prinn R. G. Fraser P. J. Simmonds P. G. Langenfelds R. L. Huang J. Cunnold D. M. Steele L. P. Krummel P. B. Weiss R. F. O'Doherty S. Salameh P. K. Wang H. J. Harth C. M. Muhle J. Porter L. W. (2008). Renewed growth of atmospheric methane. Geophys. Res. Lett. 35, L22805

[B44] Rodhe H. (1990). A comparison of the contribution of various gases to the greenhouse effect. Science 248, 1217–1219 10.1126/science.248.4960.1217 17809907

[B45] Rotthauwe J. Witzel K. Liesack W. (1997). The ammonia monooxygenase structural gene amoA as a functional marker: molecular fine-scale analysis of natural ammonia-oxidizing populations. Appl. Environ. Microbiol. 63, 4704–4712 9406389 10.1128/aem.63.12.4704-4712.1997 PMC168793

[B46] Shen X. Y. Zhang L. M. Shen J. P. Li L. H. Yuan C. L. He J. Z. (2011). Nitrogen loading levels affect abundance and composition of soil ammonia oxidizing prokaryotes in semiarid temperate grassland. J. Soils Sediments 11, 1243–1252

[B47] Steudler P. A. Bowden R. D. Mellilo J. M. Aber J. D. (1989). Influence of nitrogen fertilization on methane uptake in temperate forest soil. Nature 341, 314–316

[B48] Tamura K. Dudley J. Nei M. Kumar S. (2007). MEGA4, molecular evolutionary genetics analysis (MEGA) software version 4.0. Mol. Biol. Evol. 24, 1596–1599 10.1093/molbev/msm092 17488738

[B49] Thompson J. D. Gibson T. J. Plewniak F. Jeanmougin F. Higgins D. G. (1997). The CLUSTAL_X windows interface: flexible strategies for multiple sequence alignment aided by quality analysis tools. Nucleic Acids Res. 25, 4876–4882 10.1093/nar/25.24.4876 9396791 PMC147148

[B50] Van der Nat F. J. W. A. DeBrouwer J. F. C. Middelburg J. J. Laanbroek H. J. (1997). Spatial distribution and inhibition by ammonium of methane oxidation in intertidal freshwater marshes. Appl. Environ. Microbiol. 63, 4734–4740 16535750 10.1128/aem.63.12.4734-4740.1997 PMC1389306

[B51] Verhamme D. T. Prosser J. I. Nicol G. W. (2011). Ammonia concentration determines differential growth of ammonia-oxidising archaea and bacteria in soil microcosms. ISME J. 5, 1067–1071 10.1038/ismej.2010.191 21228892 PMC3131854

[B52] Wang J. S. Logan J. A. McElroy M. B. Duncan B. N. Megretskaia I. A. Yantosca R. M. (2004). A 3-D model analysis of the slowdown and interannual variability in the methane growth rate from 1988 to 1997 [Review]. Glob. Biogeochem. Cycles 18:B3011 10.1029/2003GB002180

[B53] Wang Z. P. Ineson P. (2003). Methane oxidation in a temperate coniferous forest soil: effects of inorganic N. Soil Biol. Biochem. 35, 427–433

[B54] Wu Y. Lu L. Wang B. Lin X. Zhu J. Cai Z. Yan X. Jia Z. (2011). Long-term field fertilization significantly alters the community structure of ammonia-oxidizing *Bacteria* rather than *Archaea* in a paddy soil. Soil Sci. Soc. Am. J. 75, 1431–1439 10.1007/s00253-011-3760-y 22159889

[B55] Xu X. Inubushi K. (2004). Effects of N sources and methane concentrations on methane uptake potential of a typical coniferous forest and its adjacent orchard soil. Biol. Fertil. Soils 40, 215–221

